# Fall supplemental feeding increases population growth rate of an endangered caribou herd

**DOI:** 10.7717/peerj.10708

**Published:** 2021-03-09

**Authors:** Douglas C. Heard, Kathryn L. Zimmerman

**Affiliations:** 1 Tithonus Wildlife Research, Prince George, British Columbia, Canada; 2 Ministry of Environment and Climate Change Strategy, Province of British Columbia, Kamloops, British Columbia, Canada

**Keywords:** Supplemental feeding, Caribou, Endangered species, Population growth, Predation risk, Nutrition

## Abstract

Most woodland caribou (*Rangifer tarandus caribou*) populations are declining primarily because of unsustainable predation resulting from habitat-mediated apparent competition. Wolf (*Canis lupus*) reduction is an effective recovery option because it addresses the direct effect of predation. We considered the possibility that the indirect effects of predation might also affect caribou population dynamics by adversely affecting summer foraging behaviour. If spring and/or summer nutrition was inadequate, then supplemental feeding in fall might compensate for that limitation and contribute to population growth. Improved nutrition and therefore body condition going into winter could increase adult survival and lead to improved reproductive success the next spring. To test that hypothesis, we fed high-quality food pellets to free-ranging caribou in the Kennedy Siding caribou herd each fall for six years, starting in 2014, to see if population growth rate increased. Beginning in winter 2015–16, the Province of British Columbia began a concurrent annual program to promote caribou population increase by attempting to remove most wolves within the Kennedy Siding and the adjacent caribou herds’ ranges. To evaluate the impact of feeding, we compared lambdas before and after feeding began, and to the population trend in the adjacent Quintette herd over the subsequent four years. Supplemental feeding appeared to have an incremental effect on population growth. Population growth of the Kennedy Siding herd was higher in the year after feeding began (*λ* = 1.06) compared to previous years (*λ* = 0.91) and to the untreated Quintette herd (*λ* = 0.95). Average annual growth rate of the Kennedy Siding herd over the subsequent four years, where both feeding and wolf reduction occurred concurrently, was higher than in the Quintette herd where the only management action in those years was wolf reduction (*λ* = 1.16 vs. *λ* = 1.08). The higher growth rate of the Kennedy Siding herd was due to higher female survival (96.2%/yr vs. 88.9%/yr). Many caribou were in relatively poor condition in the fall. Consumption of supplemental food probably improved their nutritional status which ultimately led to population growth. Further feeding experiments on other caribou herds using an adaptive management approach would verify the effect of feeding as a population recovery tool. Our results support the recommendation that multiple management actions should be implemented to improve recovery prospects for caribou.

## Introduction

Most woodland caribou (*Rangifer tarandus caribou*) populations are declining (Festa-Bianchet et al., 2011), primarily because of unsustainable predation as a result of habitat-mediated apparent competition (Seip, 1992; Wittmer, Sinclair & McLellan, 2005a; Hervieux et al., 2014). Reversing habitat-caused declines will take many decades of forest succession as the landscape reverts to a more natural age distribution that is suitable for caribou (Serrouya et al., 2011). While habitat protections like the establishment of Ungulate Winter Ranges under the British Columbia Forest and Range Practices Act help to prevent further habitat deterioration, immediate population management (i.e., treatments directly influencing population vital rates) is required to reverse population declines and prevent extirpation of small herds. Serrouya et al. (2019) demonstrated that wolf (*Canis lupus*) reduction is effective as a short-term recovery option for caribou. They also showed that population growth was greatest when wolf reduction was combined with other treatments. To further test the conclusion that multiple management actions would improve caribou recovery prospects, we examined the combined effect of wolf reduction and supplemental feeding.

Both observational and experimental evidence support the conclusion that direct mortality from predation is the main proximate factor limiting caribou population growth. Wittmer, Sinclair & McLellan (2005a) and Wittmer et al. (2005b) showed that in 15 caribou subpopulations, wolf predation was the most important cause of caribou mortality and the cause of population decline. Hervieux et al. (2014) showed that wolf reduction led to an increase in lambda, adult survival and recruitment in the Little Smokey caribou herd. Serrouya et al. (2019) showed that wolf reduction was the primary factor leading to an increase in lambda relative to controls. Food limitation and habitat loss were rejected as causes of population decline because subpopulation rates of increase were positively related to caribou density in winter foraging habitat (Wittmer, Sinclair & McLellan, 2005a), per capita abundance of old growth winter foraging habitat was not related to marrow fat (McLellan et al., 2012) and changes in caribou population growth in response to experimental population management treatments (moose and wolf reduction, caribou translocation and maternity penning) were minimally influenced by the amount of forest alteration (Serrouya et al., 2019).

Rejection of food limitation as a contributing factor to caribou population declines was based, in part, on the assumption that food limitation would occur in winter. Here, we considered the possibility that summer food may be limiting. Caribou, like many other mountain ungulates (Hebblewhite & Merrill, 2007) are generally predator sensitive foragers (Lima, 1998), trading off high quality foraging opportunities as vegetation greens up at lower elevations in order to reduce predation risk (Bergerud, Butler & Miller, 1984; Bergerud & Page, 1987; Poole, Heard & Mowat, 2000; Johnson et al., 2002; Gustine et al., 2006a; Gustine et al., 2006b; Jones et al., 2007; Ehlers, Johnson & Seip, 2016) at a time their nutritional demands are greatest and body condition is lowest (Heard, Williams & Melton, 1996; Gerhart et al., 1996; Parker, Barboza & Gillingham, 2009). We hypothesized that if summer nutrition was inadequate, then fall supplemental feeding (hereafter ‘feeding’) could at least partially compensate for that limitation, contributing to population growth by improving caribou’s body condition going into winter, increasing winter survival and leading to larger more viable calves the following spring (Veiberg et al., 2016; Gustine et al., 2017).

**Figure 1:**
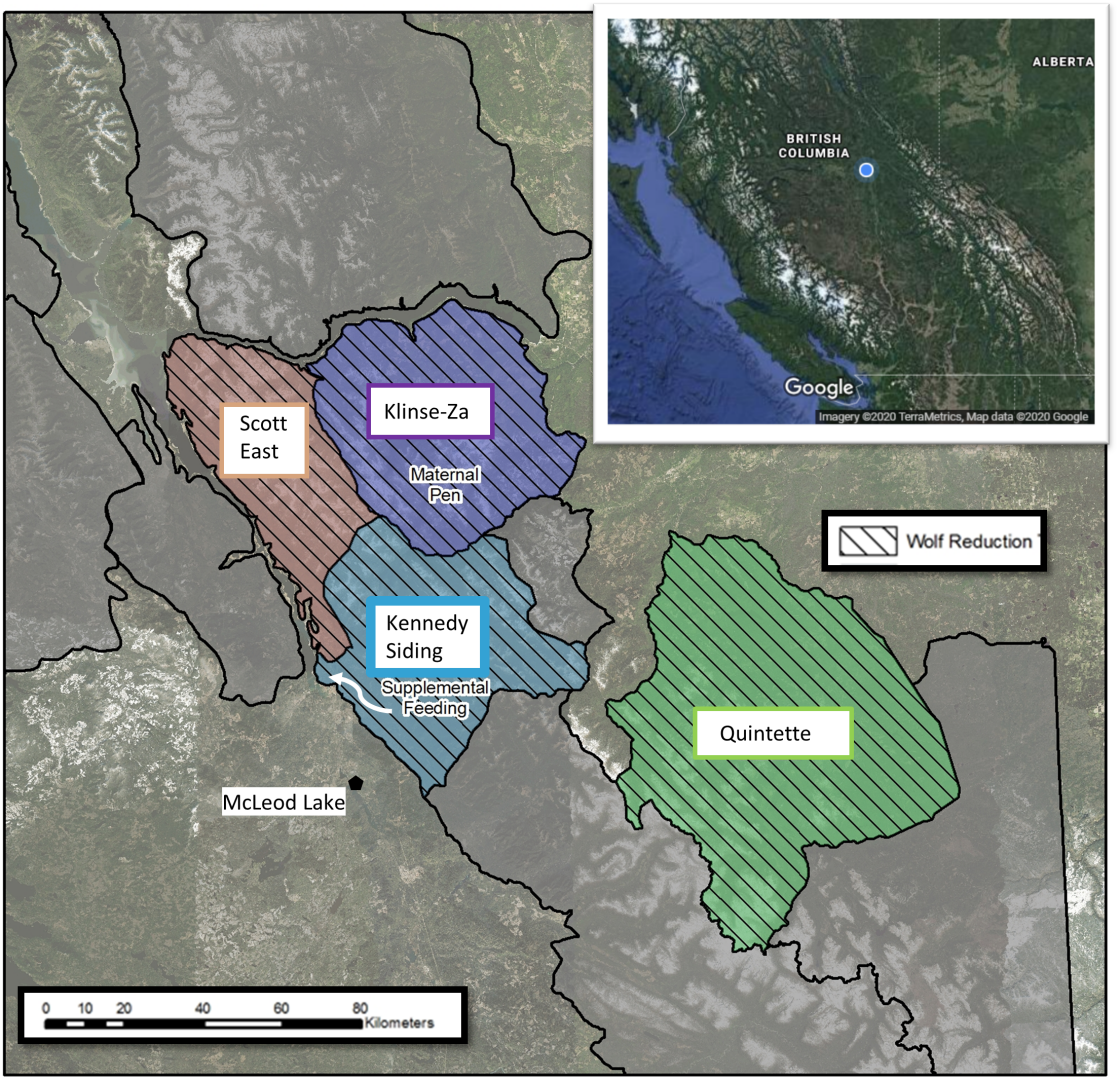
Caribou herds in central British Columbia, Canada showing the different population management treatments applied to each. Kennedy Siding herd; wolf reduction + supplemental feeding at the site indicated by the white arrow, Klinse-Za herd; wolf reduction + maternity penning, Quintette and Scott East herds; wolf reduction. Caribou in the adjacent areas outlined in black had no population management treatments. Inset map Imagery ©TerraMetrics, Map data ©2020 Google.

In 2014, many caribou subpopulations in British Columbia were declining and assessed as Endangered by COSEWIC (2014). Within the Central Mountain ecotype of caribou subpopulations (Fig. 1, Caribou Designatable Unit 8, COSEWIC, 2011), the Kennedy Siding herd declined from a high of 120 in 2007 to 41 in 2012 (Seip & Jones, 2016) and the Quintette herd had declined from 265 in 2002 to 106 in 2014 (Seip & Jones, 2018). The Klinse-Za and Scott East herds combined (also known as the Moberly/Scott herd) declined from 191 in 1997 to 16 in 2013 (Serrouya et al., 2019). In response to those declining numbers and to test our feeding hypotheses, we fed high-quality food pellets to free-ranging caribou in the Kennedy Siding herd each fall for six years starting in 2014. In addition, the Province of British Columbia began an annual program to reduce wolf density within the Kennedy Siding and adjacent caribou herd ranges (Fig. 1), and reduced wolf densities by over 80% starting in winter 2015–16 (Bridger, 2019).

Because we planned to carry out our feeding experiment on the McLeod Lake Indian Band’s traditional territory, before we began, we discussed our ideas with Alec Chingee, the McLeod Lake Indian Band’s Land Management Officer, and received his encouragement to proceed. To evaluate the effect of feeding we used a before-after-control impact design. We compared population growth rates within the Kennedy Siding herd before and after feeding began, and to contemporary growth rates in the adjacent Quintette herd, where wolf reduction was the only treatment. We also compared adult female survival and recruitment between those two herds. To make inferences about the conditions and processes that would be necessary for feeding to be effective (i.e., the inadequacy of summer nutrition and the degree to which feeding could compensate for that insufficiency) we recorded caribou body condition, and measured caribou body weight, food consumption and feeding behaviour.

Our specific objectives were to: (1) provide an ample and uninterrupted supply of food pellets to Kennedy Siding caribou in order to improve their nutritional status and increase population growth, (2) determine the population size and composition (number, age and sex of all caribou in the Kennedy Siding herd) by identifying each animal in trail camera photographs as they came to the feeders, and (3) determine if feeding had an additive effect on population growth, survival and recruitment, relative to wolf reduction alone.

## Methods

### Study area

The Kennedy Siding caribou herd’s range is characterised by mountains and rolling hills (Fig. 1) and is named after section of railroad track, called Kennedy Siding, built to load logs during the building of the nearby Williston Reservoir. Kennedy Siding caribou typically spend most of the year dispersed in relatively undisturbed sub-alpine meadows and Engelmann Spruce (*Picea engelmannii*) and Subalpline Fir (*Abies lasiocarpa*) forests (>1,400 m asl). To avoid the deep soft snow when it starts to accumulate in the mountains in fall, they descend to a small discrete area of lodgepole pine (*Pinus contorta*) forest on the southwest edge of their range, 600 m als, which has abundant terrestrial lichen (Jones et al., 2007). Most of their fall range was afforded some protection from disturbance by being designated as a 2894 ha Ungulate Winter Range under the British Columbia Forest and Range Practices Act in 2002 (Arthur, 2002). When on their fall range, caribou forage on terrestrial lichens, arboreal lichens, forbs and shrubs. Snow begins to accumulate on the ground in November and when it reaches a depth of about 50 cm (usually in mid-January), caribou move back to high elevations, where they feed on terrestrial lichens in wind-swept areas or arboreal lichens where snow is deep and supports their weight (Jones et al., 2007). The other large mammals that occupied the area and were occasionally photographed near the feeders, were moose (*Alces alces*), white-tailed deer (*Odocoileus virginianus*), elk (*Cervus canadensis*), wolf (*Canis lupus*), coyote (*Canis latrans*), grizzly bear (*Ursus arctos*), and black bear (*Ursus americanus*).

### Feeding

We provided food each fall (Fig. 1), usually starting in September and ending in mid-January, each year from 2014–15 to 2019–20. Ideally, we would have provided food during spring and summer, however it was impractical to do so because caribou are highly mobile during those times and easily disturbed when accompanied by newborn calves. In contrast, Kennedy Siding caribou used a small and easily accessible area for ∼12 weeks each fall which made feeding easier.

We contracted the McLeod Lake Indian Band to build the feeders and deliver the food pellets. Feeders were wooden boxes, approximately 60 × 120 cm with 25 cm sides and a plywood roof about 2 m above the box. The roof reduced the likelihood of pellets getting wet. Each year, either Boris Boyko or Alec Chingee delivered food to six feeders spaced over about 0.5 km^2^, usually every second day, attempting to have an ample and uninterrupted supply available. In 2018, we decided to stop feeding on 8 October, when a grizzly bear became attracted to the pellets, and resumed feeding on 13 November when we assumed that the bear had hibernated. To avoid this risk in 2019, we did not begin feeding until 3 November. We stopped feeding by 15 January each year so that we would not disrupt the traditional caribou migration back to the mountains.

To estimate pellet consumption, we accounted for the staggered arrival of caribou over time and assumed all animals remained on their fall range from the day they were first photographed at the feeders until we stopped feeding.

### Animal care

The Senior Wildlife Biologist for the Region where our research took place, and the Wildlife Veterinarian and Chair of the Animal Care Committee for the Province of British Columbia determined that no formal approval from the Animal Care Committee was required because the research was primarily an observational study that caused little or no discomfort or stress to the animals. The food pellets were manufactured by either the Wetaskiwin Coop Association in Wetaskiwin, Alberta or Hi-Pro Feeds/Trouw Nutrition Canada Inc. in Grand Prairie, Alberta. The pellets were developed specifically for caribou at the University of Alaska (Barboza & Parker, 2006) and have been widely used in other projects (e.g., Adams et al., 2019). We removed pellets from the feeders if they became wet or moldy. To check for any adverse effects of feeding, we observed caribou’s general health and noted fecal pellet consistency. To reduce disease transfer risk, we usually moved feeders to different sites among years, with no feeder at the same site for more than four years.

### Monitoring caribou numbers, condition and weight

We maintained an array of up to nine Reconyx Hyperfire motion-sensor trail cameras, focussed on either a feeder, a salt block or along a trail, so that we obtained a continuous photographic record of the entire feeding period. We examined a sample of about 20%/yr of 5 million pictures (0.7 to 1.3 million/yr), selecting pictures arbitrarily but ensuring that we examined some from all cameras and throughout the feeding period. For each caribou, we reviewed as many images as necessary from different orientations, to count the number and position of all points on each antler. In addition, we determined the caribou’s sex and age (i.e., calf or adult), and occasionally other unusual markings (e.g., exceptionally white legs or ears) which we were confident allowed us to distinguish all adults. Calves did not always have unique antlers, so we usually identified them by their close association with their mother. K Zimmerman and technicians C Bratley, B Heard, J Colbourne, D Breault and C Ryley examined photographs and D Heard reviewed and confirmed that every caribou identified was unique. The high degree of variation in antler morphology allowed for individual identification, but the requirement to examine antlers from different perspectives and the variation in picture quality prevented us from automating the identification process. We referred to caribou less than one year old as male calves or female calves and older caribou as males or females.

We believed that our maximum counts in January following fall feeding (i.e., January 2015 for the fall feeding from October 2014 to January 2015) represented a total count of the population (i.e., we identified all of the caribou that were in the Kennedy Siding herd each year), because a) trail cameras photographed all of the radio-collared females that came to the feeders, (three to ten per year; 2015=3, 2016=4, 2017=6, 2018=9, 2019=10, 2020=9), b) the cumulative count asymptoted well before caribou left in January, and c) a random sample of 22 females captured on their winter range in March (three to six per year; 2015=6, 2016=3, 2017=4, 2018=4, 2019=5), and recognisable by their antlers, never included individuals that we had not identified the previous fall (radio-collared caribou numbers and locations from the Province of British Columbia, Knowledge Management Branch). We knew the composition data was not perfect because in fall 2019 we found two more males than we had counted in fall 2018 (Table 1), possibly because of a photo interpretation error (e.g., in determining calf sex, or missing caribou that was on the fall range but not recorded) or because of immigration. The only evidence that the Kennedy Siding herd was not a closed population was when one radio-collared female that came to the feeders in fall 2014, remained on her summer range and did not return in fall 2015, and died before fall 2016. All other radio-collared females returned in subsequent years (*n* = 41, years combined) and we never photographed a radio-collared female from any of the adjacent herds even though there were several radio-collared females in all adjacent herds throughout the duration of our study. Fidelity of males was unknown because no males were radio-collared.

**Table 1:** Number of caribou at Kennedy Siding each fall from 2014–15 to 2019–20. In fall 2019 we found 2 more males than we had counted in fall 2018, possibly because of a photo interpretation error (e.g., in determining calf sex, or missing caribou that was on the fall range but not recorded) or because of immigration.

YEAR	2014–15	2015–16	2016–17	2017–18	2018–19	2019–20
Females	26	26	27	32	36	44
Female calves	3	2	7	6	11	3
Males	16	19	18	24	23	32
Male calves	4	3	11	3	7	8
Total caribou	49	50	63	65	77	87
*Adjustments for human caused mortality and emigration of one female in 2014–2015:*						
Females	−1	−1	0	−1	0	0
Female calves	0	0	0	0	0	0
Males	-1	0	0	-1	0	0
Male calves	0	0	0	0	0	0
Adjusted total caribou	47	49	63	63	77	87

Females (%)	62	58	60	57	61	58
Calves (%)	14	10	29	14	23	13

**Figure 2:**
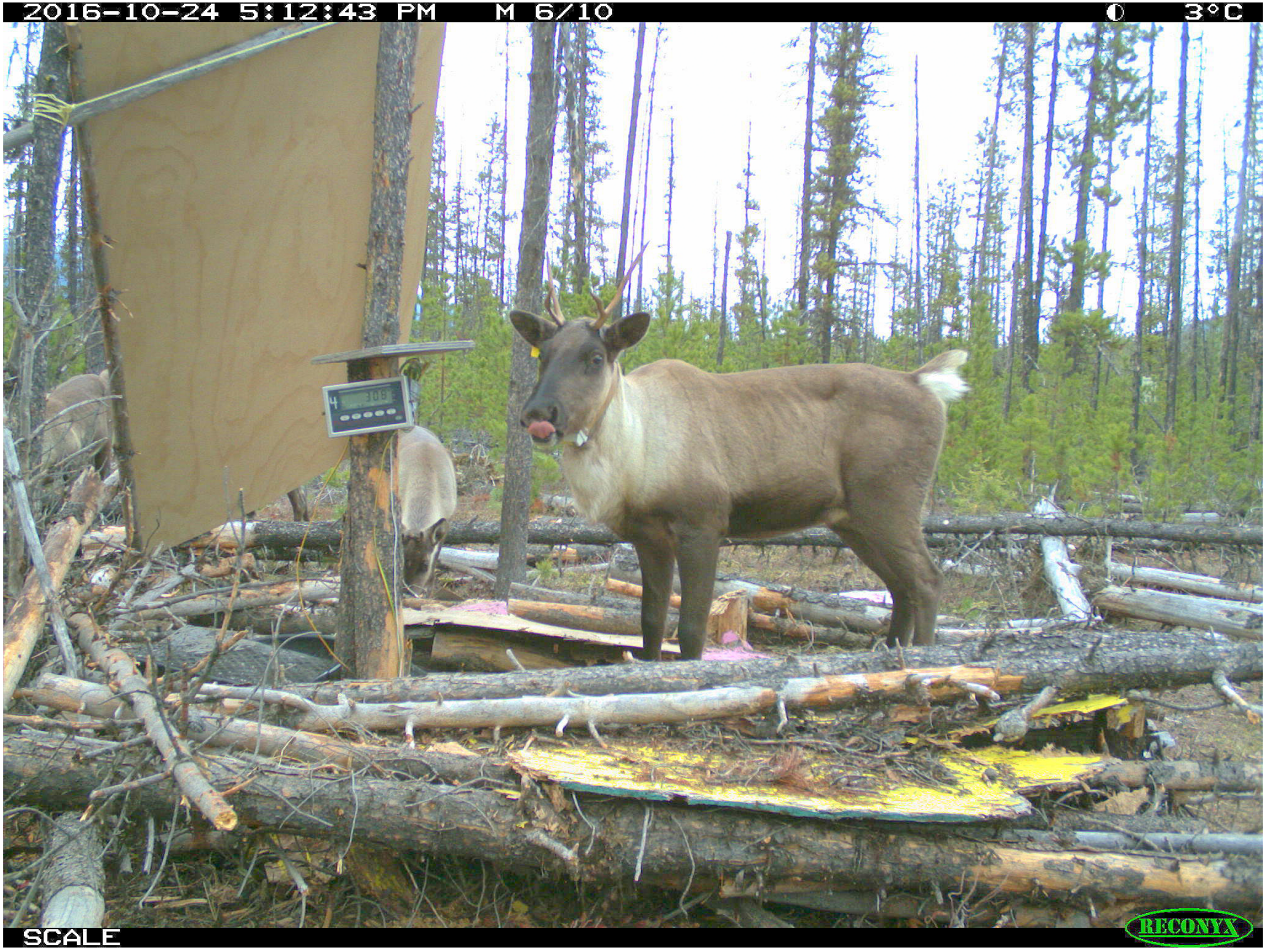
Trail camera photograph of a Kennedy Siding radio-collared cow caribou with visible ribs on the platform scale with the readout displaying a weight of 308 pounds. Photo credit: Doug Heard.

When we first detected a caribou, we recorded whether their ribs were visible in the trail photograph and we used that as an index of their body condition. Because nutritional condition is accurately assessed by live weight (Taillon et al., 2011), we attracted caribou onto a platform scale (Fig. 2) using salt or food pellets as described by Bassano et al. (2003), with trail cameras positioned to photograph the individual caribou and record the weight on the scale’s digital display. Because ten pictures were taken every time motion triggered the camera and the weight displayed on the scale after the caribou had left, we were able to account for the weight of any snow or debris on the platform.

We indexed caribou activity at feeders by hour of the day (Pacific Daylight Time) from September (October in 2014) through January 2014–15 to 2019–20, based on the kernel density estimate of the number of pictures taken per hour using the geom_density_ridges function in r (R Development Core Team, 2019). Pictures were from one arbitrarily selected camera that operated continuously through the feeding period. Images that were not triggered by caribou were removed. The index was based on 70,494, 132,975, 205,421, 166,716, 164,333, 131,682 pictures per year from 2014–15 to 2019–20 respectively.

### Lambda calculations

All data are posted on figshare.com and available here: https://figshare.com/s/1bff14f6907dde8ca9d9 https://figshare.com/articles/dataset/KS_for_r_xlsx/13248515 and https://figshare.com/articles/dataset/Kennedy_Siding_caribou_activity_index/12801083.

All analyses were done using R software version 3.6.3 (R Development Core Team 2019). The R code for all analyses is presented on GitHub https://github.com/heard-hub/PJV4.

We calculated lambda (*λ*) for the Kennedy Siding herd prior to the start of feeding in fall 2014 based on three spring aerial survey counts between 2007 and 2012, and spring recruitment (R) counts (percent calves) and radio-collared female mortality (M) from Seip & Jones (2016) using the RM model, *λ*=(1-M)/(1-R), where R = (number of calves/2)/[(number of adults*0.59)+(number of calves/2)] (Hatter & Bergerud, 1991; DeCesare et al., 2012; Serrouya et al., 2017). Lambda was female-based because M was the mortality rate of radio-collared females, R was the percent of female calves (assuming a 50:50 calf sex ratio) and 0.59 was the proportion of adults that were female (based on the results of this study). Lambda estimates, based on the RM model, have been shown to correlate with aerial survey counts in caribou (Serrouya et al., 2017). In 2008, when there was no estimate of R, we used the mean R from 2007 and 2009 to estimate lambda. We modeled the number of caribou each year (t) from 2007 to 2014 by multiplying population size in year t-1 by the *λ* for year t, using the starting population size in 2007 that minimised the sum of the squared differences between the three aerial survey counts and the modeled estimate for those years. We calculated five lambda values from the six total counts (2015 to 2020), incorporating the four caribou killed by people (three probably shot and one vehicle collision) and one female that emigrated (i.e., by remaining on her summer range), into the herd’s growth rate for that year.

We estimated lambda for the Quintette caribou herd from 2008 to 2020, using the RM model with spring recruitment and mortality estimates from Seip & Jones (2017), Seip & Jones (2018), Pelletier & Seip (2019) and A Pelletier, pers. comm. (2020), except for 2016 when there were no recruitment or mortality data. We did not use population estimates based on aerial survey counts because changes in caribou distribution rendered the 2019 and 2020 estimates unreliable (Pelletier & Seip, 2019 and A Pelletier, pers. comm., 2020). We estimated lambda for 2016 by averaging the modelled lambda from 2014 to 2015 and total count lambda from 2014 to 2016. We modeled the number of caribou each year (t) from 2008 to 2020 by multiplying population size in year t-1 by the *λ* for year t, using the starting population size in 2013 that minimised the sum of the squared differences between the 2013 to 2018 aerial survey counts and the modeled estimate for those years.

We used spring wolf density estimates reported by Bridger (2019) for the Quintette herd and for the adjacent Pine River Landscape Population Unit, which included both the Kennedy Siding and Klinse-Za herd ranges. We plotted the pre-removal wolf density estimate against the pre-treatment mean caribou lambdas, and annual estimates of wolf density and caribou lambdas by treatment thereafter, assuming spring wolf density would affect lambda over the subsequent year.

## Results

### Arrival timing, number and composition of the Kennedy Siding caribou herd

**Figure 3:**
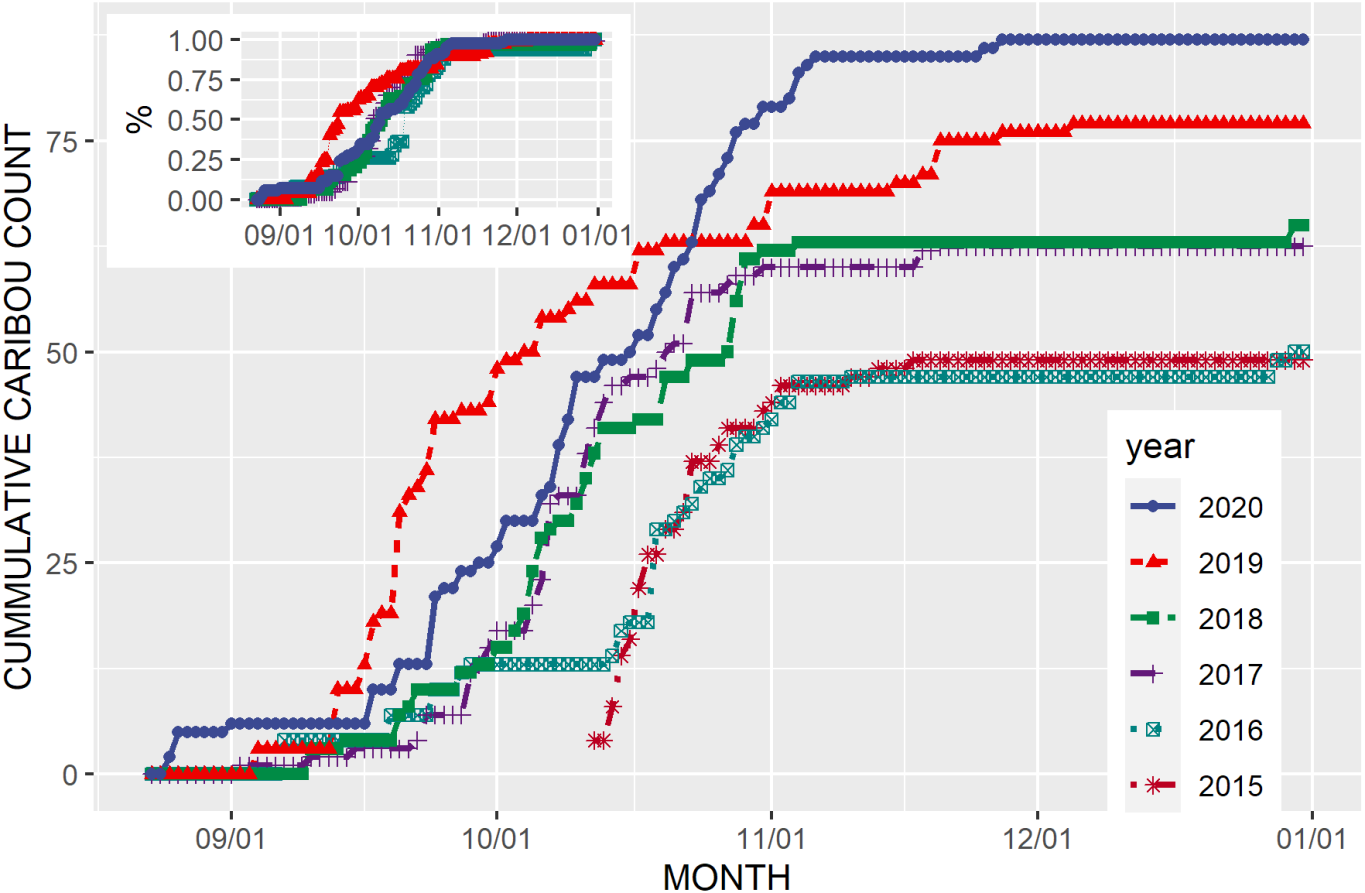
Cumulative number of caribou by date on the fall range of the Kennedy Siding herd each year from 2015 to 2020. Inset figure is the cumulative proportion of caribou by date each year from 2015 to 2020.

Most caribou arrived on their fall range during the October rut (Fig. 3, Bergerud, 1971)). Females and males showed the same pattern of arrival over time with the median annual arrival dates between 7 and 19 October except in 2018–19, when presumably due to early snowfall in the mountains, the median date was about three weeks earlier (14 September) than in the other five years. By 15 September between five and 13% of the caribou had arrived, and by 1 November, between 84% and 95% had arrived.

The number of caribou that we identified in the Kennedy Siding herd increased from 49 to 87 during the six years with population management (2014–15 feeding only, 2015–16 to 2019–20 feeding + wolf reduction; Table 1, Fig. 3). Adult sex ratio averaged 69 males:100 females (59% females), ranging from 62 to 75:100. Of the 68 calves observed, 32 were female (47%) and calves averaged 17% of the population, ranging from 10% in 2016, to 30% in 2017. We recovered the carcasses of 4 caribou near the feeders. One was killed in a vehicle collision. One had definitely been shot and left intact and 2 were probably shot, because they were butchered, and parts of the carcass had been removed.

### Population growth in relation to management treatments

**Figure 4:**
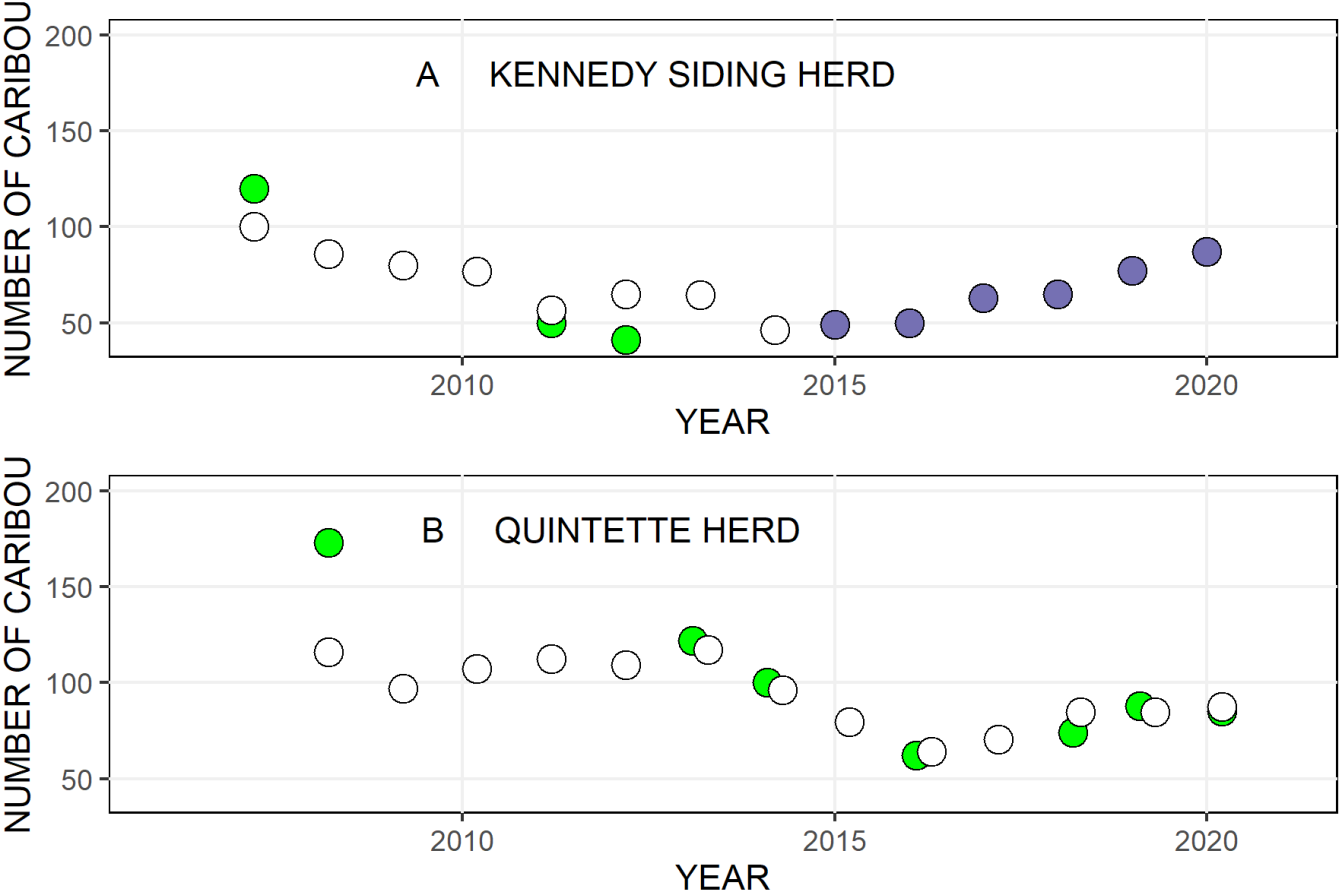
The number of caribou in the Kennedy Siding and Quintette caribou herds in central British Columbia from 2007 to 2020. (A) Kennedy Siding herd. (B) Quintette herd. Open circles are population estimates based on the RM model, green dots are population estimates based on aerial survey counts and purple dots show census counts from fall trail camera photographs. Overlapping Quintette points were slightly offset for clarity.

**Figure 5:**
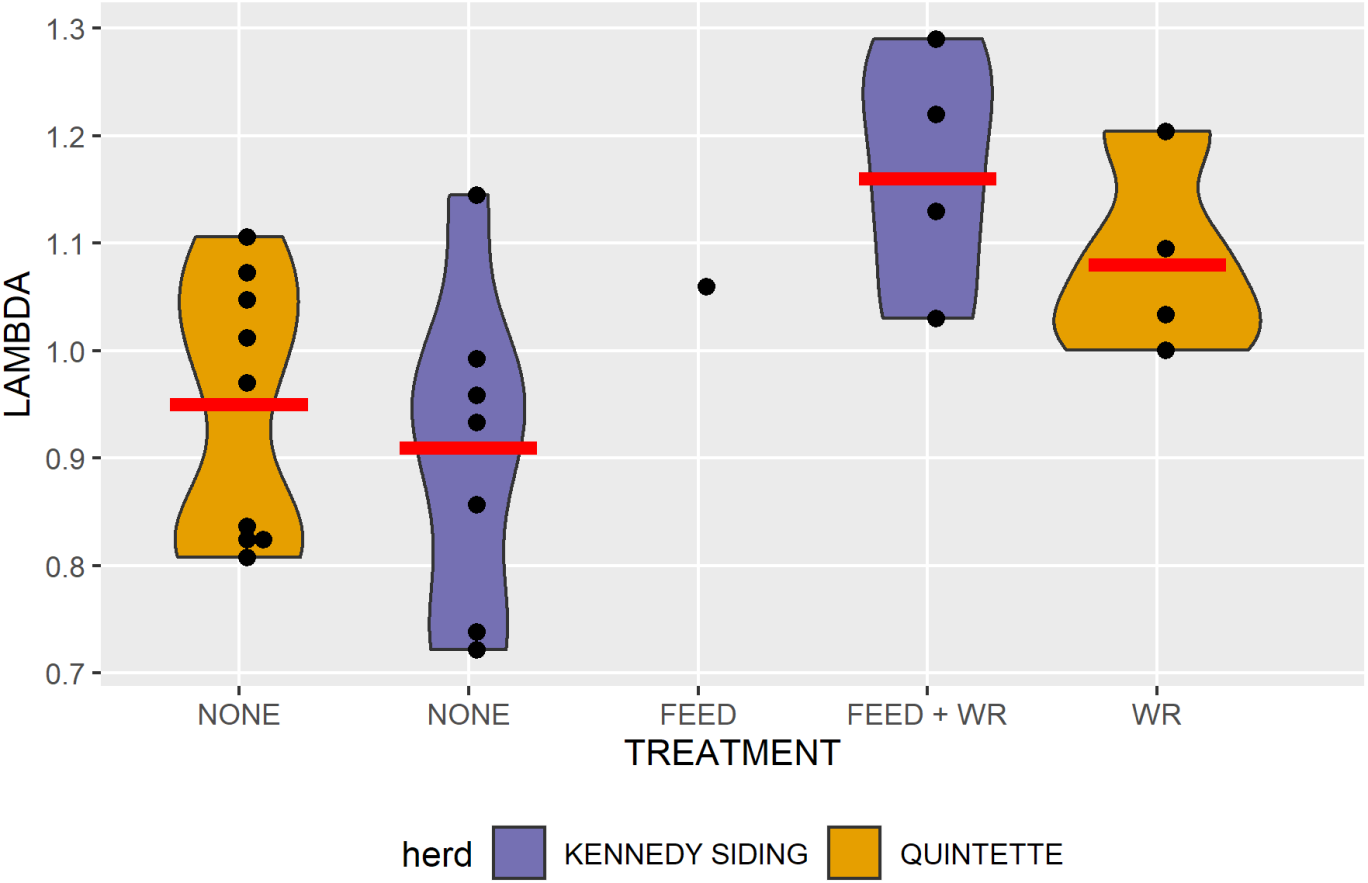
Population growth rates when there was no management treatment, feeding only, feeding plus wolf reduction and wolf reduction only applied to the Kennedy Siding and Quintette caribou herds.

Kennedy Siding herd aerial survey counts from 2007 to 2012 indicated that the herd declined from 120 to 41 animals (*λ* = 0.81), while the lambda estimates from the RM model suggested *λ* = 0.91 (Figs. 4, 5). After the first year of feeding, from 2015 to 2016, when feeding was the only treatment *λ* = 1.06. Over the four years we provided food, and wolf densities were reduced (2016 to 2020), the geometric mean growth rate increased to *λ*=1.16/yr (Table 2, Figs. 4, 5). Although annual growth rates ranged from 1.03 to 1.29 over those four years, because we had a total count of the population each year, there was no uncertainty associated with the 4-year mean growth rate.

Lambda estimated from aerial survey counts of the Quintette herd between 2008 and 2016 was 0.89, while lambda estimated from the RM model suggested a similar but more gradual decline of *λ* = 0.95 (Figs. 4, 5). We estimated lambda from 2015 to 2016 at 0.81, from the average of the modelled lambda from 2014 to 2015 (*λ* = 0.83) and aerial survey lambda from 2014 to 2016 (*λ* = 0.79). Even though the 2019 and 2020 aerial survey counts were considered unreliable they were similar to the RM modelled estimates. Between 2016 and 2020, when wolf densities were reduced, the geometric mean lambda using the RM estimates was *λ* = 1.08/yr (SE=0.041, *n* = 4, Figs. 4, 5).

Feeding appeared to have an incremental effect on population growth in addition to the effect of wolf reduction. Lambda after the first year of feeding, when feeding was the only treatment, was higher than the Kennedy Siding trend in prior years and higher than the control population for that year (*λ* = 1.06 vs *λ* = 0.91 and *λ* = 0.81 respectively). Over the subsequent four years, feeding appeared to increase population growth by 8%/yr. The probability that lambda from wolf reduction alone (*λ* = 1.08/yr) was as high as lambda for wolf reduction + feeding (*λ* = 1.16/yr) was 0.086% (one-sample *t*-test).

**Figure 6:**
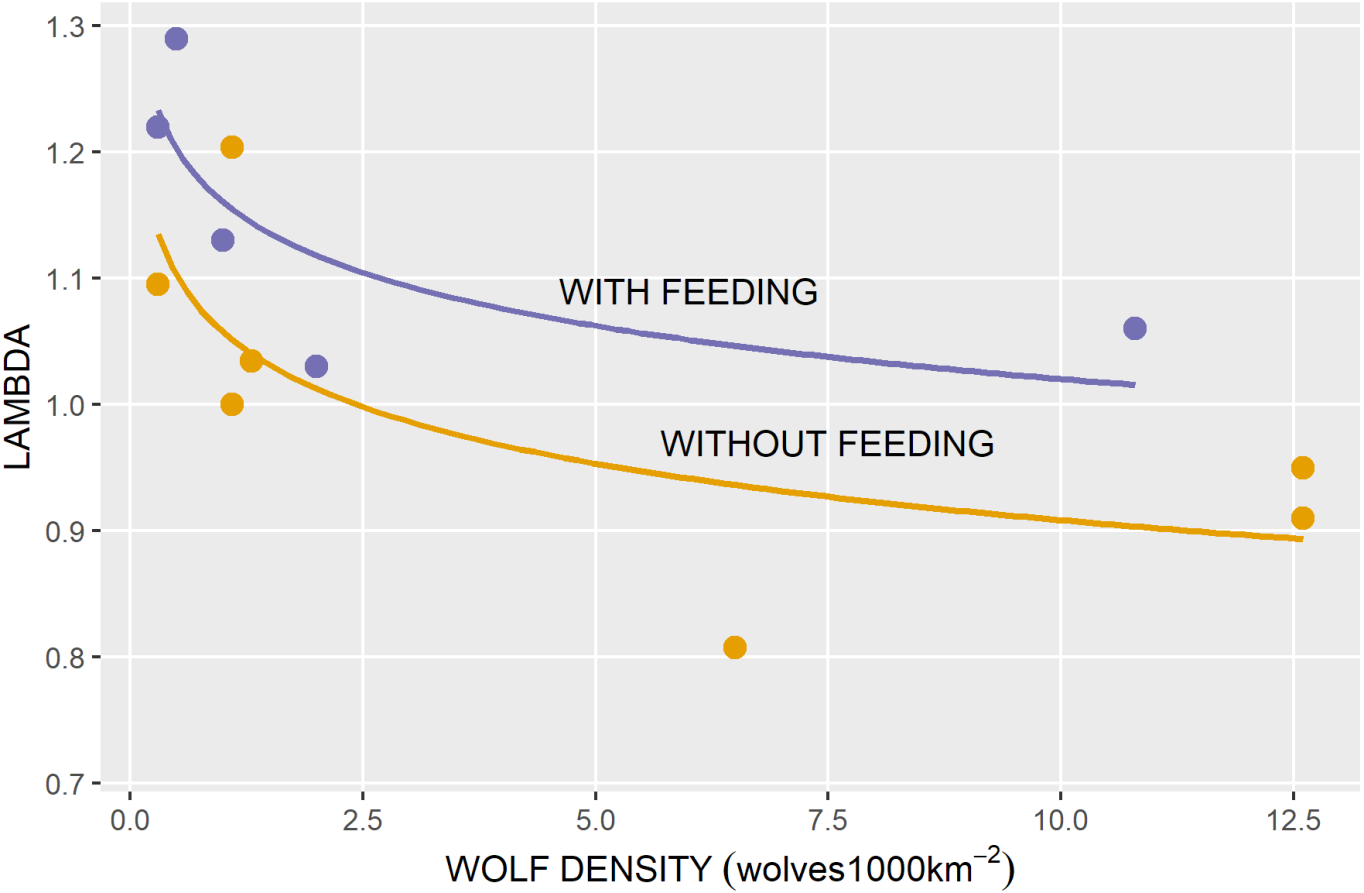
Annual population growth rates (lambda) in relation to wolf density for caribou herds with and without feeding.

Wolves on both the Kennedy Siding and Quintette ranges were reduced to a mean of 0.95 wolves/1000 km^2^ from between 10.8 and 12.6 wolves/1,000 km^2^ (Fig. 6, Bridger, 2019). The effect of wolf density reduction on lambda appeared to be non-linear, with the greatest impact occurring when wolf densities were reduced to <2 wolves/1000 km^2^, with feeding appearing to have a constant additive effect across all wolf densities (Fig. 6).

**Table 2:** Survival and population growth rates (Lambda) of caribou in the Kennedy Siding herd in central British Columbia, Canada, during the years when supplemental food was provided.

YEAR	2015 to 2016	2016 to 2017	2017 to 2018	2018 to 2019	2019 to 2020
Female survival	92.9	100	94.1	97.3	93.6
Male survival	100	81.8	82.8	88.5	106.7
Adult survival	95.7	91.8	88.9	93.7	98.7
Lambda	1.06	1.29	1.03	1.22	1.13

### Survival and recruitment

Having an annual total count of caribou by age and sex allowed us to calculate survival rates between years as the proportion of adults at Kennedy Siding in year *t* +1 divided by the number of calves + adults in year *t*. The natural survival rate therefore included calves, after they were identified at Kennedy Siding at about six months old. Annual female survival over the four years with feeding + wolf reduction averaged 96.2% (SE = 1.49, *n* = 4, Table 2).

Assuming female calves survived at the same rate as females from fall to spring, female calf recruitment averaged 16.5%/yr from 2016 to 2020. If female calves had a lower survival rate than females, then female calf recruitment would be <16.5%/yr and female survival >96.2%/yr. Between 2007 and 2014 when the herd was declining, female recruitment averaged 8.7%/yr and female survival 82.4%/yr.

Between 2015 and 2020, an average of 36% of females were accompanied by a calf in fall. That percentage peaked in alternate years (Table 1). Alternate year calf production was also the case for individual females. Forty-one percent of the radio-collared females were accompanied by a calf. In 65% (35 of 54) of the occasions when a radio-collared female was present two years in a row, she was accompanied by a calf in only one of those years, which was far more frequent than expected if being accompanied by a calf in fall was random. If producing a calf that survived until fall was random, we would expect only 48% of the females to have calves in alternate years, given the probability of having a calf in year one and not in year two = 24% (.41 ×(1–.41)) plus 24% for females without a calf in year one but with one in year two (Chi-square test = 6.12, *df* = 1, *p* = 0.013). On only six of those 54 occasions (11%), were females accompanied by a calf both years, and no females were accompanied by a calf three years in a row (*n* = 33).

Mean percentage of calves among years in the Kennedy Siding herd (19.8%, SE = 3.82, *n* = 4 years) was essentially the same as in the Quintette herd (20.6%, SE = 1.49, *n* = 4, 2017-2020). With no difference in recruitment between herds, the higher lambda at Kennedy Siding was related to higher mean annual female survival; 96.2% in the Kennedy Siding herd versus 88.9% in the Quintette herd (SE = 4.67, *n* = 4).

### Body weight and condition

**Figure 7:**
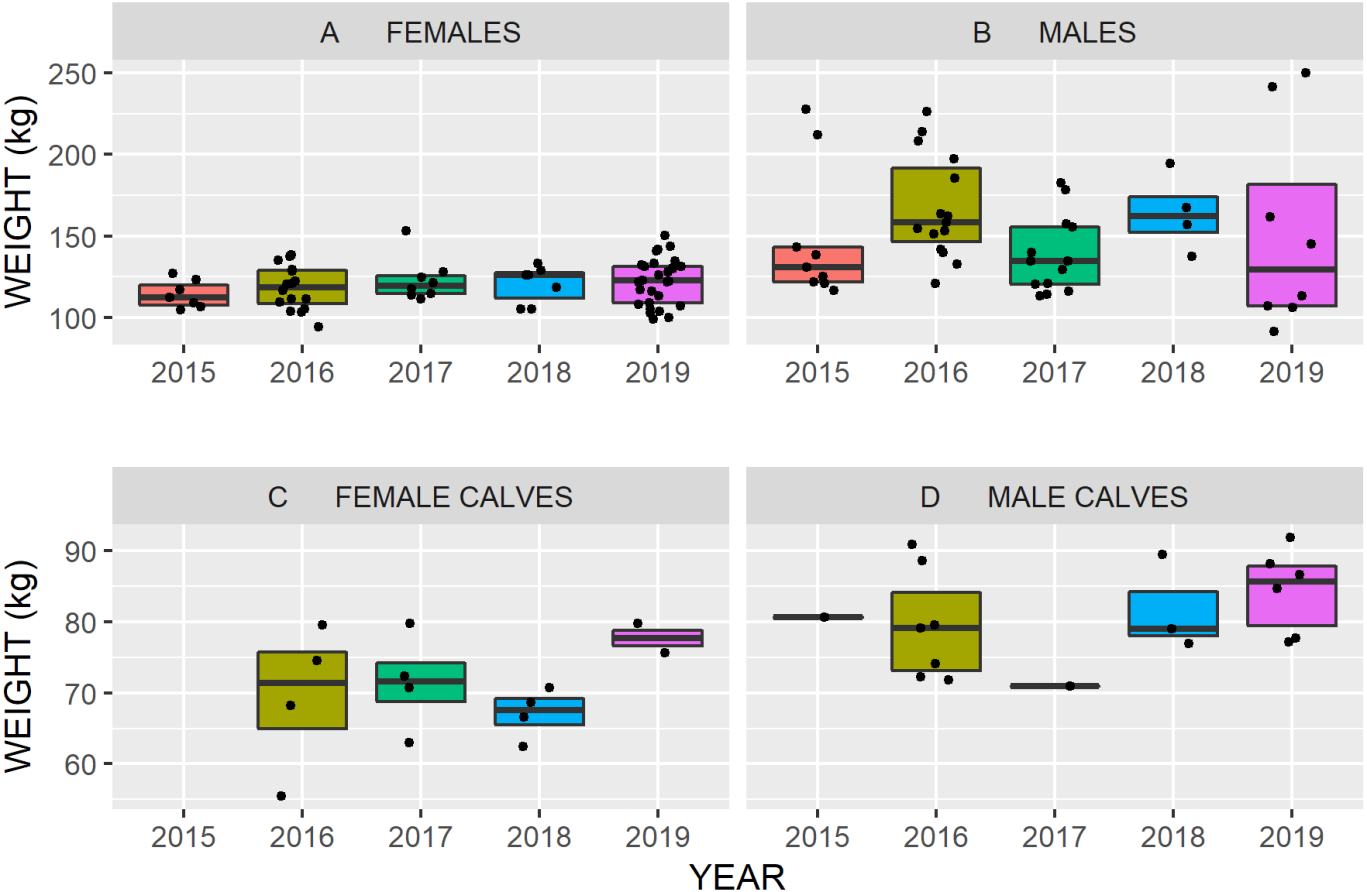
Body weights (black dots) of Kennedy Siding caribou by age, sex and year from fall 2015 to 2019. The median weight is the thick horizontal black line and boxes enclose the inner quartile range of weights (i.e., 50% of the weight observations). When caribou were weighted more than once, we plotted the mean weight. The *y*-axis range differs between the upper and lower panels

We determined weights of 147 caribou over five years (Fig. 7). Mean weight of male calves (81.1 kg, SE = 1.62, *n* = 18) was 10 kg heavier than female calves (70.5 kg, SE = 1.92, *n* = 14). Female weights averaged 120.3 kg (SE = 1.61, *n* = 66) and males 153 kg (SE = 5.4, *n* = 49).

**Figure 8:**
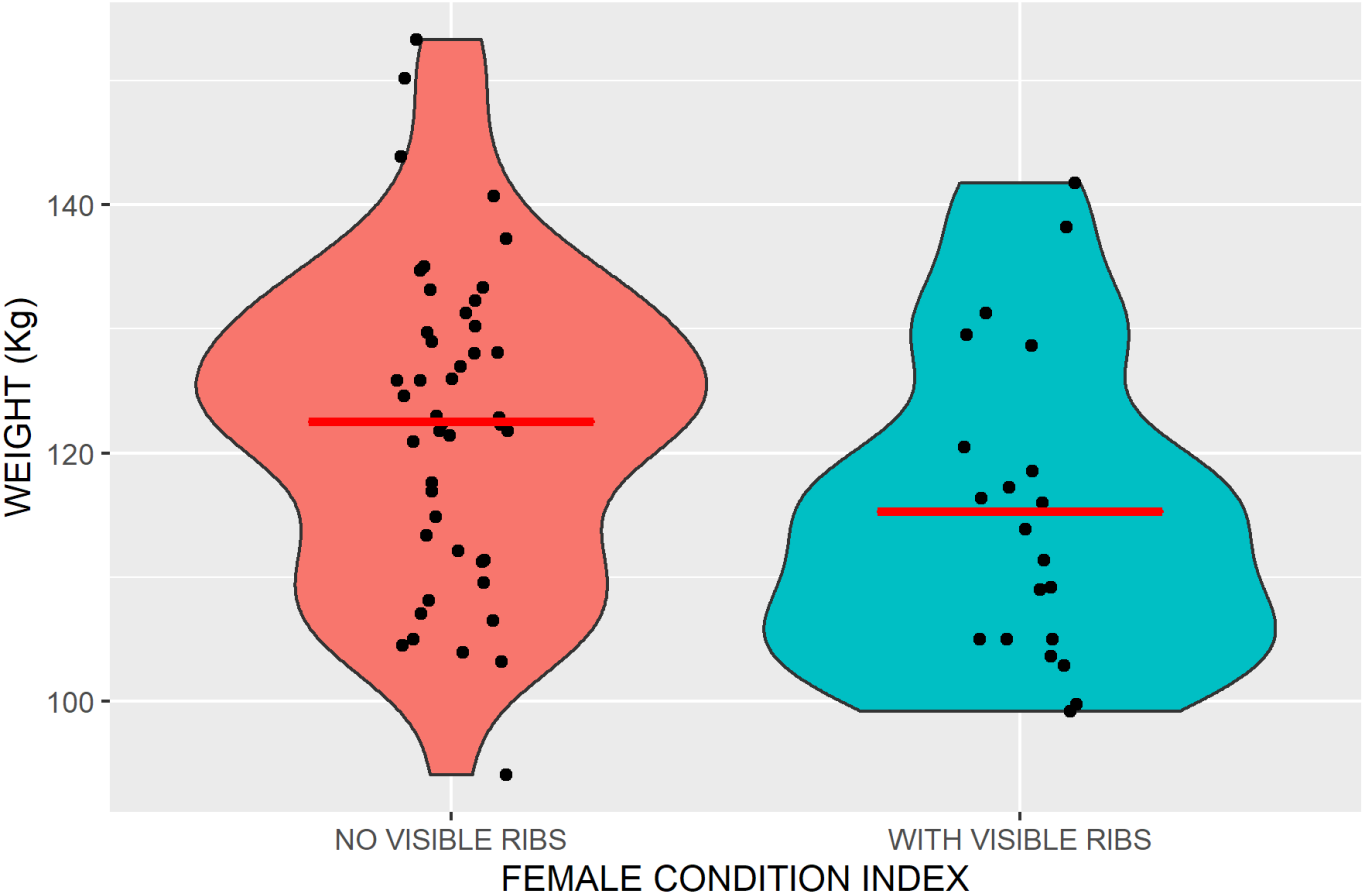
Violin plots outlining the kernel probability density of Kennedy Siding female caribou weights with and without visible ribs. The width of the shaded area represents the proportion of weights (black dots) located there. The red bar marks the mean weight.

Seven percent of 63 calves (range over six years 0 to 15%), 29% of 125 males (range over six years 11 to 47%) and 27% of 187 females (range over six years 19 to 36%) had visible ribs (mean of six annual means). In females, the presence of visible ribs seemed to accurately reflect condition as the mean weight of 21 females with visible ribs was 115.3 kg (SE = 2.7), whereas the mean weight of 45 other females was 122.6 kg (SE = 1.92, Fig. 8). If females could not compensate for lactation costs, then the weight of females that had lactated, would have been lighter than those females that did not lactate. The mean weight of females with a calf at heel was the same as those that were not accompanied by a calf (120 kg) but the weight of females without a calf was not an accurate index of lactation costs because females without a calf would have included both young females, that would be lighter and less likely to have conceived and lactated, and females that had lactated for only part of the summer. Lactation demands may have been reflected in body condition, as 55% of females with a calf (35 of 56) had visible ribs while only 15% of other females did (19 of 131).

Over the entire study, average rate of weight gain for 12 caribou weighted twice over period of >1 month was 103 gm/d for females (SD = 118, *n* = 6) and 94 gm/d for males (SD = 123, *n* = 6). Over 76 days, the minimum length of time food was provided, 103 g/c/d would result in a 7.8 kg weight increase (103 g/c/day*76 days), more than the weight difference between caribou with and without visible ribs.

### Food Provided

Depending on the year and their arrival date, caribou in the Kennedy Siding herd had access to food pellets for between 76 and 106 days. During that time, we provided about 1.1 kg pellets/c/d (range among four years 2015 to 2019; 0.9 to 1.3 kg/c/d). Those values represent only a crude estimate of consumption. Even though all ages and both sexes appeared to feed together with only occasional evidence of any social displacement, some caribou (a proportion we could not quantify) appeared at the feeders more often than others suggesting that consumption was not equally spread among individuals. In addition, while caribou clearly ate most of the food, some unknown amount was eaten by ravens and bears, and some food was discarded when it got wet.

**Figure 9:**
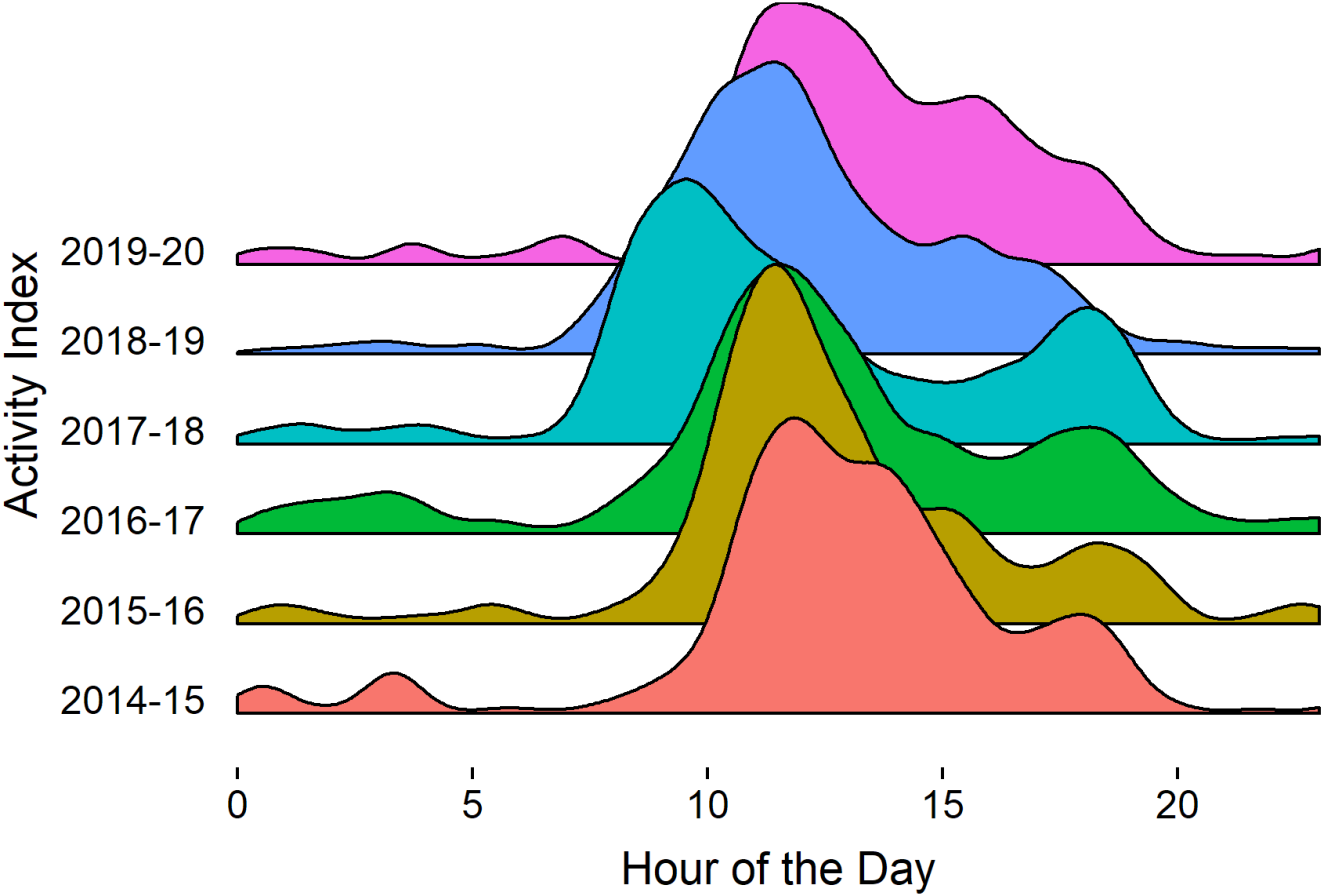
Index of Kennedy Siding caribou activity at feeders by hour of the day based on the kernel density estimate of the number of pictures taken per hour. Indices were based on 70,494, 132,975, 205,421, 166,716, 164,333, 131,682 pictures per year from 2014–15 to 2019–20 respectively. Sunrise ranged from 06:58 on 21 Sep to 09:38 on 21 Dec and sunset from 19:12 on 21 Sept to 16:44 on 21 Dec (https://sunrise-sunset.org/ca/mackenzie/2018/9) Pacific Daylight Time.

We used the relative number of photographs taken by one trail camera focused on a feeder from Sept through January, by hour of the day, as an index of feeding activity. Caribou activity at feeders occurred mostly in daylight, with 73% of the activity from 09:00 to 17:00 and 40% between 10:00 and 13:00 (Fig. 9). When not at the feeders, caribou had access to, and presumably foraged on, terrestrial lichen and other natural food, as they did prior to the start of feeding.

## Discussion

Feeding appeared to have an incremental effect on population growth above the effect of wolf reduction, based on before-after and contemporary control comparisons, of population growth rates among treatments. Comparisons from a single year may be subject to random variation, especially, as in this study, when numbers are small. None-the-less, over the first year of feeding, when feeding was the only treatment, lambda was greater than it was in prior years in the Kennedy Siding herd and greater than in the Quintette herd for the same year. Over the subsequent four years, feeding appeared to have an incremental effect of 8%/yr on population growth over wolf reduction alone.

Effect size can also be measured as the change in lambda (Δ*λ*). Change in lambda from feeding + wolf reduction was 0.25 (1.16–0.91), greater than the change in lambda from wolf reduction alone, not only for the Quintette herd where Δ*λ* = 0.13 (1.08–0.95) but also for all of the other caribou herds where wolves were reduced, as reported in Serrouya et al. (2019), Scott East Δ*λ* = 0.13, À la Pêche Δ*λ* = 0.13, Little Smoky Δ*λ* = 0.06, South Selkirk Δ*λ* =  − 0.09).

In the Klinse-Za herd, from 2014 to 2018, where wolf reduction was accompanied by maternal penning *λ* = 1.14 and Δ*λ* = 0.28 (Serrouya et al., 2019), the population growth rate was almost the same as feeding + wolf reduction. Feeding, which occurred in spring, may have been a contributing factor in the Klinse-Za herd growth because, (1) as Adams et al. (2019) showed, penned females and calves received nutritional benefits from their consumption of food pellets, and (2) the penned lactating caribou are the ones most likely to benefit from the receiving supplemental food.

High growth rate in the Kennedy Siding herd was consistent with Serrouya et al. (2019) who showed that multiple recovery actions applied simultaneously resulted in the highest population growth rates for caribou. The most dramatic increase in lambda was at wolf densities <2/1000 km^2^. This density is consistent with the Federal recovery strategy recommending wolf density be reduced <3/1000 km^2^ (Environment Canada, 2014) and Serrouya et al. (2019) who showed that high intensity management actions (e.g., reducing wolves to a very low density) may be required for recovery.

For fall feeding to have been effective, summer nutrition must have been inadequate, supplemental food consumption must have allowed caribou to compensate for that insufficiency, and any increase in body condition must have had cascading impacts on future vital rates. In an environment with abundant natural food females should be in peak condition in fall even if they have lactated throughout the summer (Ouellet et al., 1997; Couturier et al. 2009; Parker, Barboza & Gillingham, 2009). This was not the case in the Kennedy Siding herd, as each year we observed many caribou, especially females with calves, in relatively poor body condition based on their light weight and visible ribs. At least some caribou were unable to put on as much fat as would be expected under ideal summer foraging conditions. As Gerhart et al. (1997) noted, only females that lactated over summer provide information of nutritional adequacy during summer and early autumn. The overrepresentation of alternate year calf production is consistent with what one would expect if some females were not able to recover from lactation costs (Cameron, 1994; Gerhart et al., 1997). Pregnancy rates would not likely be influenced by feeding in the year feeding occurred.

Caribou appeared to have eaten enough of the supplemental food to have increased their body condition. Average consumption of 1.1 kg pellets/c/d for at least 76 days, represented a substantial nutritional addition to their diet given that caribou fed the same ration in captivity, with no access to other food, consumed between 2.2 and 3 kg/c/d (Parker, Barboza & Stephenson, 2005; Barboza & Parker, 2008; Thompson & Barboza, 2014). Also, 76 days was a relatively long addition to a typical 100 d summer weight gain period (Denryter et al., 2020) especially given that those 76 days occurred when natural foods were of low quality because of plant senescence and caribou’s appetite drive is still high (Barboza & Parker, 2008).

An increase in body condition could improve vital rates over subsequent months and years through many processes (White, 1983; Hamel et al., 2009; Parker, Barboza & Gillingham, 2009; Bårdsen & Tveraa, 2012; Ballesteros et al., 2013; Gustine et al., 2017; Paoli et al., 2019). We observed a higher mean annual survival rate for females in the Kennedy Siding herd relative to Quintette herd females (96.2% vs 88.9%) but mean percentage of calves was essentially the same in both herds (19.8% vs 20.6%). That result was consistent with Wittmer et al. (2005b) who showed that population trends in mountain caribou were correlated with female survival rate but not with calf recruitment. If caribou in better condition take fewer foraging risks, it could result in the higher female survival we observed. Other potentially cascading effects of improved fall body condition, but that we did not measure, are (a) greater calf birth mass (Bårdsen & Tveraa, 2012), higher calf viability (Adams et al., 2019; Paoli et al., 2019), lower risk of dying from predation (Mattisson et al., 2016; Mumma et al., 2019), and earlier birthing date/shorter gestation (Gustine et al., 2017; Paoli et al., 2019). Supplementary feeding has been used to improve late winter condition of many ungulate species (Putman & Staines, 2004; Peterson & Messmer, 2007; Jones et al., 2014). In reindeer specifically, Ballesteros et al. (2013) showed that winter feeding led to higher population growth rate, higher productivity and heavier calves. We were unaware of any other attempts to feed only in the fall.

Although the results were consistent with our hypothesis that summer nutrition may limit population growth, they do not explain why nutrition appeared to be inadequate. Our reasoning remains tenable that at the landscape scale, caribou reduced predation risk by remaining on their high elevation winter ranges rather than risk foraging in spring at lower elevations with nutritious greening-up vegetation and high wolf densities (Bergerud & Page, 1987; Gustine et al., 2006a; Jones et al., 2007; Ehlers, Johnson & Seip, 2016). At the foraging site scale, caribou appeared to reduce predation risk by foraging primarily during the day, a time when wolves are least active (Fig. 9, Shores et al. 2019). If caribou are extremely risk averse, then nutritionally stressed individuals would be reluctant to increase foraging risk and may not have a great influence on the proportion of caribou that are in poor condition in predator diets, as observed by McLellan et al. (2012), especially if there are relatively few animals in poor condition. It is also possible that food availability and summer nutritional condition declined for other reasons. For example, climate warming may reduce the abundance (Paoli et al., 2019) or quality (Gustine et al., 2017) of important forage species. Warming that results in earlier green-up, shifting peak nutrient availability away from peak energy demands, may lead to nutritional stress in females in some populations as it did in Greenland caribou Post & Forchhammer (2008) even though that effect was not found elsewhere (Tveraa et al., 2013; Veiberg et al., 2016; Gustine et al., 2017; Paoli et al., 2019; Mallory et al., 2020).

The conclusion that unsustainable predation was primarily responsible for caribou population declines and reduction in predation was responsible for the greatest increase in lambda (Wittmer et al., 2005b; Serrouya et al., 2019), does not preclude other factors, like summer nutrition, from also limiting population growth (McLellan et al., 2012). Our results suggest that feeding had an incremental effect on population growth above the effect of wolf reduction. Feeding may be even more effective as caribou population size, density at the foraging scale and intraspecific competition for food all increase (Hurley, Hebblewhite & Gaillard, 2020). It is also possible that the combined effect of feeding and predator reduction may increase over time or under different ecological conditions as observed in snowshoe hares (*Lepus americanus*) and Arctic ground squirrels (*Spermophilus parryii*) (Krebs et al., 1995; Hodges, Krebs & Sinclair, 1999; Karels et al., 2000).

### Management implications

Our experiment needs to be repeated on other herds for a more thorough assessment of its effectiveness and generality, and to reduce the likelihood that our results were attributable to random variation, or something unique to the Kennedy Siding herd. If further work provides similar results, then feeding has potential as a recovery option because it is effective immediately, minimally invasive, applicable as a sole treatment or in combination, low risk, low cost and based on comments we received from the public and members of the McLeod Lake Indian Band, socially acceptable. In addition to its nutritional and demographic benefit, feeding may facilitate an estimate of population size and composition. Annual cost of feeding at Kennedy Siding, where there was road access, was about $7000/caribou (CDN), considerably less than costs estimated for other management treatments like wolf reduction ($26,000/caribou) or maternity penning ($148,000/caribou; Johnson, Mumma & St-Laurent, 2019).

If accessible and used repeatedly, feeding sites may increase risks from hunting, poaching, predation, vehicle collisions, disease transfer (Murray et al., 2016), and result in adverse effects from changes to movement patterns (Peterson & Messmer, 2007; Jones et al., 2014). We attempted to reduce those risks by erecting signs to remind people that in the area occupied by caribou: 1) shooting and hunting was prohibited, 2) the recommended speed limit was 30 km/hr and 3) the McLeod Lake Indian Band requested that Band members not exercise their tradition right to hunt caribou. We moved feeders among years and after 2017 placed all feeders on the same side of the road to reduce caribou’s vulnerability to vehicle collisions. We stopped feeding by 15 January each year so that we would not disrupt the traditional caribou migration pattern. We did not know of any predation deaths, but over the six years of this study there were three shooting deaths and one fatal vehicle collision. Despite those losses, the population grew at the highest rate recorded for woodland caribou. In predator free environments, the maximum growth rate for *R. tarandus* is *λ* = 1.3 (Heard, 1990). The growth rate we documented (*λ* = 1.16) can be used as an obtainable goal for short term caribou recovery. Actions to further increase growth rate need to focus on improving neonatal survival, because at 96%, there is little room to increase survival of caribou older than six months (Table 1).

Feeding may be an appropriate management action for other small and declining caribou herds, especially where summer range conditions have been shown to be inadequate (e.g., as measured by poor body condition, lack of lactational compensation, or low pregnancy rates), where caribou are predictable enough to have a high likelihood of encountering food, where caribou are familiar with eating pellets (e.g., Klinse-Za and Columbia North where many caribou spent time in the maternal pens; (Serrouya et al., 2019), or where an alternative population estimation technique would be beneficial. Feeding could be considered as a general technique to promote recovery of endangered species, even those primarily limited by predation.

## Conclusions

1. Fall feeding appeared to have an additional affect on population growth rate over the affect of wolf reduction alone.
2. Some caribou, mainly lactating females, were in relatively poor body condition in the fall.
3. Feeding increased the body condition of caribou and had cascading beneficial demographic affects.
4. Our results were consistent with Serrouya et al. (2019) in that management treatments used in combination and intensively resulted in higher population growth rates.
5. Replicating our experiment using an adaptive management approach would clarify the effect of feeding as a population recovery tool.

##  Supplemental Information

10.7717/peerj.10708/supp-1 Supplemental Information 1 Relationship between presence of snow and the arrival of caribou at Kennedy Siding

## Additional Information and Declarations

### Field Study Permissions

The following information was supplied relating to field study approvals (i.e., approving body and any reference numbers): Before we began, we discussed our ideas for this research with Alec Chingee, the Band’s Land Management Officer, receiving both verbal and written encouragement to proceed.

### Data Availability

The following information was supplied regarding data availability: All raw data are available at Figshare: - Heard, Douglas (2020): KS_for_r.xlsx. figshare. Dataset. https://doi.org/10.6084/m9.figshare.13248515.v1
- Heard, Douglas (2020): Kennedy Siding caribou activity index. figshare. Dataset. https://doi.org/10.6084/m9.figshare.12801083.v1
All analyses were done using R software and the code for all analyses is available at GitHub Heard, Douglas & Zimmerman, Kathryn (2020): PJV4. GitHub. Code. https://github.com/heard-hub/PJV4.
