# Supplementary material for: Fall supplemental feeding increases population growth rate of an endangered caribou herd
Source: PeerJ. 2021 Mar 9;9:e10708. doi: 10.7717/peerj.10708 (PMC7953878; doi:10.7717/peerj.10708)
Supplement: Supplemental Information 1 [file peerj-09-10708-s001.pdf]

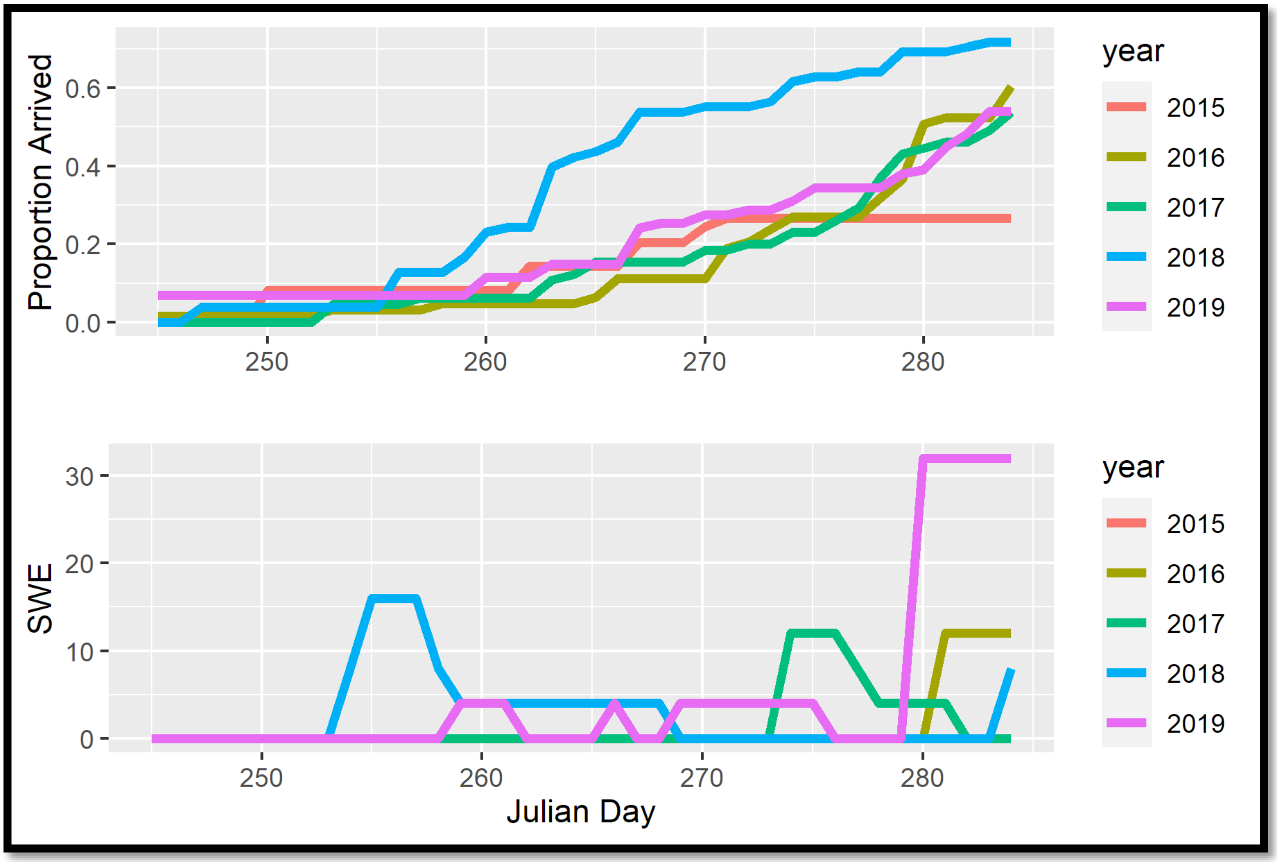

Top graph: Proportion of caribou that had arrived at Kennedy Siding each year (2015-2019) between day 244 (1 September) and day 285 (12 October) showing that a higher proportion of caribou had arrived after day 255 in 2018 than in the other years.

Bottom graph: Presence of snow on the ground, based on the snow water equivalent (SWE) measures obtained from ORNL DAAC (Daymet Software Version 3.0; Daymet Data Version 3.0 Thornton; P.E.; M.M. Thornton; B.W. Mayer; Y. Wei; R. Devarakonda; R.S. Vose; and R.B. Cook. 2016. Daymet: Daily Surface Weather Data on a 1-km Grid for North America; Version 3. ORNL DAAC; Oak Ridge; Tennessee; USA. http://dx.doi.org/10.3334/ORNLDAAC/1328). We extracted the daily SWE for 3 mountain locations where there were radioed cows in mid-September for 2015 through 2019. Lines connect the daily summed SWE for those sites. On day 255 the percent of caribou that had arrived was about 5% in all years, but in the 10 days following the early (day 255) 2018 snowfall, the percent of caribou that had arrived in 2018 increased to 44% but to only 13% in the other years. That 31% difference persisted through mid-October.
